# The Syn-Q study: detection of cutaneous phosphorylated alpha-synuclein in Parkinson’s disease progression: a trial protocol

**DOI:** 10.3389/fneur.2026.1823991

**Published:** 2026-04-14

**Authors:** Jourdan H. Parent, Todd Levine, Roy Freeman, Christopher H. Gibbons

**Affiliations:** 1 CND Life Sciences, Scottsdale, AZ, United States; 2 Department of Neurology, Beth Israel Deaconess Medical Center and Harvard Medical School, Boston, MA, United States

**Keywords:** biomarker, Parkinson’s disease, phosphorylated alpha-synuclein, REM sleep behavior disorder, skin biopsy

## Abstract

**Background:**

Parkinson’s disease (PD) is a neurodegenerative condition with progressive phosphorylated alpha-synuclein (P-SYN) deposition. Skin biopsies are a sensitive and specific source of tissue to detect intra-axonal P-SYN in patients with PD. Further, isolated REM sleep behavior disorder (iRBD) is a prodromal condition with a high risk of phenoconversion to PD. The objective of the Synuclein-Quantification (Syn-Q) study is to quantify cutaneous P-SYN across a range of PD severity and in iRBD and measure changes in P-SYN over time.

**Methods:**

After consent, participants with iRBD and PD across Hoehn and Yahr stages 1, 2, 3 will complete neurological and cognitive evaluation, motor and olfactory assessments, orthostatic vitals, and questionnaires. Skin biopsies (3 mm diameter and 3–4 mm depth) will be taken from distal leg 10 cm above the lateral malleolus, distal thigh 10 cm above the lateral knee, and posterior cervical region 3 cm lateral to the C-7 spinous process with quantitation of cutaneous intra-axonal P-SYN. Participants will return for follow-up visits to repeat study procedures at 6, 12, and 18 months following the baseline visit. This study is funded by the Michael J. Fox Foundation and is registered on Clinicaltrial.org (NCT06621602).

**Expected results:**

We anticipate that patients with more severe stages of PD will have higher levels of quantitative P-SYN. We also predict that increases in P-SYN over time will be associated with worsening disease severity.

**Discussion:**

In patients with PD, the detection and quantification of P-SYN is a critical step to supporting clinical trials that seek to alter the natural history of disease. Skin biopsies offer a more accessible, repeatable and quantifiable approach to monitoring phosphorylated alpha-synuclein compared to cerebrospinal fluid. This study will define the longitudinal rates of P-SYN change in patients with PD from prodromal to advanced disease.

**Clinical Trial Registration:**

https://clinicaltrials.gov/study/NCT06621602

## Introduction

Parkinson’s disease (PD) is a progressive neurodegenerative disorder with variable clinical phenotypes that include motor, autonomic and cognitive symptoms. The range of clinical features creates challenges to the early and accurate diagnosis of PD (1). Worldwide, over 11 million people are living with PD, and this is expected to increase to ~25 million people by 2050 (2) making it the most common movement-related neurodegenerative disorder (3). At autopsy, PD is pathologically characterized by the deposition of phosphorylated alpha-synuclein (P-SYN) within the central and peripheral nervous systems as aggregated proteins called Lewy bodies and Lewy neurites (4, 5).

Recently, the use of skin biopsies to detect peripheral Lewy neurites has bridged autopsy-level disease confirmation with an in-vivo diagnosis (6). Skin biopsy detection of P-SYN is one of several novel diagnostic advances in patients with PD (7–9). Skin biopsies have the advantage of being well-tolerated, relatively non-invasive and easily repeatable (10, 11). A recent prospective, blinded, cross-sectional multicenter study demonstrated over 92% sensitivity and over 96% specificity in detecting P-SYN from skin biopsies of patients with PD (10). The potential utility of P-SYN as a biomarker of disease holds broad applicability, ranging from improved clinical decision making, early detection and quantitation of change over time (12–14). Further, developments in early detection of PD could be bolstered by efforts to investigate P-SYN accumulation prodromal syndromes. Indeed, individuals with the prodromal syndrome isolated REM sleep behavior disorder (iRBD) have a high risk of phenoconversion to PD (15) and early detection and quantification of P-SYN in these individuals may better predict the likelihood of conversion to PD. In addition, the reliable quantification of cutaneous P-SYN has the potential to serve as a biomarker of target engagement in disease-modifying therapies.

The goals of the Synuclein-Quantification (Syn-Q) study are to (1) define rates of cutaneous P-SYN detection across a well-characterized group of patients with either prodromal iRBD or PD across a range of severities, (2) quantify the amount P-SYN detected from each of three biopsies to define the topographical distribution and, (3) measure rates of P-SYN change over time. Successful completion of this study will establish a foundational understanding of the natural progression of cutaneous P-SYN in patients with established or prodromal PD.

## Methods

### Study design and cohort

This is a multi-center, blinded, prospective, longitudinal, observational study to evaluate phosphorylated alpha synuclein pathology in cutaneous tissues in individuals with prodromal disease (iRBD) and PD across a range of disease (Hoehn and Yahr stage I, II & III). This study aims to enroll 75 subjects diagnosed with PD with 25 subjects enrolled in each Hoehn and Yahr stage (I, II, and III), totaling 75 participants (16). Their disease state will be assessed by the International Parkinson and Movement Disorders Society Unified Parkinson’s disease rating scale (MDS-UPDRS) completed at the baseline visit. Additionally, 25 subjects diagnosed with iRBD, confirmed by polysomnogram (PSG), without evidence of PD, will be included based on their medical history. A total of 100 subjects will be enrolled. Subjects will be evaluated at baseline, 6 months, 12 months, and 18 months (+/− 30-day window).

#### Inclusion criteria for PD cohort

1. Male and female 50–85 years of age.
2. Confirmed diagnosis of PD by MDS clinical diagnostic criteria for Parkinson’s disease (17).
3. Stage I, II, or III only as defined through the Hoehn & Yahr Scale in the OFF state which is a staging system measuring the progression and severity of PD symptoms (16). Stage I is defined as unilateral involvement only, Stage II is defined as Bilateral involvement without impairment of balance and Stage III is defined as mild to moderate bilateral disease, some postural instability, and physical independence. Mild–moderate stages were chosen for monitoring progression into more severe disease staging.

#### Inclusion criteria RBD cohort

1. Male and female 50–85 years of age.
2. Confirmed diagnosis of iRBD confirmed by polysomnogram (PSG) (15); without symptoms of other neurodegenerative diseases.

#### Expert panel review

An expert panel of 2 movement disorder specialists and one neurologist with expertise in neurodegenerative disease will review all baseline and follow-up medical records, including inclusion criteria, questionnaires, structured examinations, and special investigations. Participants will be categorized or recategorized as iRBD, PD Stage I, PD Stage II, or PD Stage III (i.e., fulfilling diagnostic criteria with all information present or, if data are missing, as being highly probable to meet criteria for the specific diagnosis). Participants who do not fulfill diagnostic criteria for iRBD or PD will be categorized as indeterminate. Conclusions will be reached by consensus. Results of skin biopsies will be redacted from the medical records. In the event that this redaction is incomplete, the expert panel will not use skin biopsy results to form a diagnostic conclusion.

#### Exclusion criteria for all subjects

1. Any history of a prior negative test for synuclein pathology in cerebrospinal fluid or skin.
2. Clinical evidence of severe vascular disease (history of ulceration, poor wound healing, vascular claudication).
3. History of allergic reaction to local anesthesia needed for skin biopsies.
4. Use of blood thinners (aspirin or Plavix alone is allowed).
5. Significantly impaired wound healing, history of scarring or keloid formation.
6. Concurrently enrolled in a disease-modifying clinical trial.

### Study visits and participant data collection

#### Trial synopsis

Approximately 25 sites will participate in recruiting 75 individuals diagnosed with Parkinson’s disease and 25 patients with iRBD in an 18-month longitudinal study where skin biopsies will be performed at 3 sites on each patient at 6-month intervals (baseline, 6, 12, and 18 months). Detailed quantified neurological examination, cognitive evaluation, medical history, and questionnaires will be performed at each visit. Additional biomarkers, imaging, and clinical information (if available) will be obtained for the purpose of determining disease progression for PD cohort and phenoconversion to clinically apparent synucleinopathy for the iRBD cohort. All testing will be repeated at all visits to define any changes to clinical diagnosis (clinical phenoconversion or progression). Skin biopsies will be repeated at the 6- and 12- and 18-month follow-up visits to determine the rate of P-SYN accumulation over time and the rates of nerve fiber degeneration within punch skin biopsies.

Study visit will include

1. Review of enrollment forms (inclusion and exclusion criteria).
2. Informed consent.
3. Review of medical history and current medications.
4. Quantified neurologic examinations: All participants will have standardized neurologic examinations using published methods. The neurological examinations will be performed by the treating physicians. The specific examinations will include:

a MDS-UPDRS (Parts I, II, III & IV) (18).

- Part III motor assessments will be performed in the “OFF” state, preferably with the last dose of Levodopa taken the night before. After conducting the MDS-UPDRS Part III, medication can be taken. (In order to reduce ‘OFF’ time for the participant, it is suggested that the MDS-UPDRS part III should be conducted as soon after consent as possible). The remainder of study visit would be conducted while patient is in the “ON” state. For those participants who are being treated with Deep Brain Stimulation, the stimulation will be turned off for the MDS-UPDRS part III assessment.

b Hoehn and Yahr scores.

5 Smell Testing: All subjects will complete the University of Pittsburgh Brief Smell Identification Test (BSIT) a 12-item, self-administered scratch and sniff test designed to measure olfactory function (19).
6 Orthostatic vitals to be measured with the participant supine after 5 min and then standing after 3 min.
7 Blood Plasma collection:

c One 4 mL EDTA tube for genetic testing for inherited forms of PD (LRRK2, GBA1, PRKN, PINK1, and SNCA) which is collected at the baseline visit only.
d One 10 mL EDTA tube collected at all visits for blood-based biomarkers (plasma p-tau-217, neurofilament light chain, glial fibrillary acidic protein). as exploratory outcomes.

8 Three millimeter diameter and 3–4 millimeter depth punch skin biopsies taken from the posterior cervical area 3 cm lateral to the C-7 spinous process, distal thigh 10 cm above the lateral knee, and distal leg 10 cm above the lateral malleolus after local analgesia with 1% lidocaine (9).
9 Cognitive/Neurological Testing and Questionnaires: All participants will complete the following questionnaires/assessments:

e European Quality of Life Instrument (EQ-5D & VAS) (20)
f SLUMS cognitive assessment
g Parkinson’s Disease Questionnaire-39 (PDQ-39) (21)
h Orthostatic Hypotension Questionnaire (OHQ) (22)
i REM sleep behavior disorder questionnaire (RBDSQ)
j Scales for Outcomes in Parkinson’s Disease-Autonomic (SCOPA-AUT)
k Geriatric Depression Scale-short form (GDS)

10 Collection of medical records for the last 3 years including any imaging (DaTscan, MRI, PET, SPECT, neurologic testing or genetic testing)

All assessments and blood collection will be repeated once every 6 months, with the exception of genetic testing which takes place only once at the baseline visit.

### Standardization of data and tissue collection

To minimize potential variability in study outcomes, all tissue and clinical data will be obtained using standard methods that we have utilized in multiple prior studies (10, 23).

#### Skin biopsy samples

Punch skin biopsies 3 mm in diameter and 3–4 mm in depth will be taken from all subjects at the distal leg 10 cm above the lateral malleolus, the distal thigh 10 cm above the lateral knee, and the posterior cervical region 3 cm lateral to the C-7 spinous process. Biopsy locations are selected based on prior publications that demonstrate differential phosphorylated alpha-synuclein deposition in proximal and distal locations across the different synucleinopathies (24, 25). The Brain-First, Body-First theory of P-SYN deposition would suggest that patients with RBD prior to other manifestations of PD might have greater distal P-SYN deposition (26). The three sites we have selected provide maximal sensitivity and specificity across all synucleinopathies (9). Local analgesia will be provided using lidocaine with epinephrine (1:100,000). Skin biopsy samples do not require sutures, and band aids will be provided for wound care. Skin biopsy specimens will be fixed in Zamboni paraformaldehyde solution in pre-labeled tubes, placed into a standard shipping pack, and shipped overnight for central processing. At the central processing site, biopsies will be washed and placed in a 20% glycerol cryoprotectant solution.

#### Skin biopsy processing and immunostaining

After storage in cryoprotectant solution for at least 24 h, skin samples will then be sectioned into 50 μm thick samples using a cryostat. Tissue will be immunostained with antibodies against protein gene product 9.5 (PGP9.5: a pan-axonal marker to identify nerve fibers) and with antibodies to phosphorylated alpha-synuclein using free-floating sections in our standard protocols. Six skin biopsy specimens from each biopsy will be dual immunostained with rabbit anti-protein gene product 9.5 (Cederlane: 1:1,000) and phosphorylated alpha-synuclein (Wako: 1:1,000), mounted on glass slides as previously reported (9). An immunofluorescent microscope will perform an initial review of slide quality. In all experiments, positive and negative controls are run to confirm the results.

#### Biopsy analysis

All biopsies will be evaluated in a blinded fashion using standard protocols with slides randomly intermixed with other blinded studies to maintain data integrity (27). Cutaneous axons are identified by the antibody binding to protein gene product 9.5. Nerve fiber subtypes are defined by their pathological characteristics within dermal substructures, including sudomotor (sweat gland) fibers, pilomotor fibers (within piloarrector muscles), vasomotor nerve fibers surrounding blod vessels, nerve bundles, intra-epidermal nerve fibers and the subepidermal plexus (28). Pathologist reading will include a semi-quantitative analysis of each biopsy site. This reading includes the number of positive cutaneous dermal structures for each tissue section for each biopsy and also accounts for the intensity of P-SYN staining (29). All slides will also undergo complete digitization (VS200, Evident). A series of optical images will be acquired at 2 μm intervals through the depth of the section as a Z-stack (VS200, Evident). A custom-developed digital image analysis package (NerValence, CND Life Sciences) integrated with Oncotopix software (Visiopharm, Hørsholm, Denmark; Version 2023.09.3.15043) was designed and optimized specifically for objective identification and quantification of cutaneous nerve fibers and phosphorylated alpha-synuclein (P-SYN) deposits within digitized immunofluorescent slide images. This artificial-intelligence (AI) augmented algorithm will be used to detect and quantify cutaneous intra-axonal P-SYN deposits (30).

Quantitation of P-SYN (um^2^ of P-SYN per mm^2^ of tissue analyzed) will be reported for each biopsy specimen. The amount of P-SYN will be noted for each cutaneous dermal sub-structure (blood vessels, nerve bundles, pilo-arrector muscles, sweat glands, and sub-epidermal plexus) as previously reported (23). Intra-epidermal nerve fiber density (IENFD) will be calculated using standard methodology for immunostained nerve fibers, reported as nerve fibers/millimeter (31). Pathological review and grading of P-SYN deposition for all tissue sections will follow strict blinding measures, including slide randomization, random number generation, and blinding between pathology reviewers and study staff (9). Results of the AI-generated data will be reviewed against pathologist confirmed results in all biopsies.

#### Definition of “positive biopsy results”

Only biopsies expressing P-SYN co-localized within a peripheral nerve axon (confirmed using immunostaining for anti-PGP9.5 and anti-P-SYN) will be considered positive. Any equivocal section will be considered negative. Any positive result indicates a positive case (e.g., if one of 3 biopsies is positive, the case is positive).

#### Clinical data capture

All study questionnaires, examination scores, and demographic information, will be acquired using electronic data capture. Ancillary data (including prior medical records, test results, and polysomnography) will be uploaded into the electronic data capture system.

### Blinding and review of data

#### Study blinding

The clinical and biopsy data will be blinded and maintained separately. Pathology slides will be randomized using numbers assigned by the Electronic Data Capture System (EDC). Blinded slides will be batch processed in combination with multiple other blinded studies to ensure all staff and pathology readings are blinded to study, visit and disease state. Unblinding will occur only at the time of database lock.

### Objectives

This is a prospective longitudinal study of individuals with PD and prodromal RBD to establish cutaneous P-SYN deposition as a stable, reliable quantitative measure that changes in parallel with disease progression. The results of this study will provide the basis for establishing cutaneous P-SYN as a biomarker of response to disease-modifying therapeutic interventions and a possible surrogate biomarker for outcomes in clinical trials of disease-modifying therapies. The primary objectives of this study are:

1. To determine rates and patterns of P-SYN detection in iRBD and PD across Hoehn and Yahr stages I, II and III.
2. To measure the change quantity and pattern of cutaneous P-SYN deposition over time.
3. To enhance pathological detection and quantitation of P-SYN using rapid, high resolution, confocal microscopy combined with a novel AI-augmented pathology protein detection system.

### Secondary objective

We predict that AI-enhanced quantitative detection of cutaneous P-SYN will provide an accurate, continuous outcome measure that will correlate with disease stage and severity. Whole slide imaging with extraction of representative neural structures, target stain detection, segmentation, stain quantification, and pattern recognition will be performed using deep learning algorithms (NerValence, CND Life Sciences). We anticipate that results will be correlated with MDS-UPDRS scores, cognitive assessments, Hoehn and Yahr Stage, and daily levodopa dose.

### Statistical analysis and blinding

#### Expected outcomes

We hypothesize that cutaneous P-SYN will increase by >10% per year in patients with PD. We will confirm our ability to quantify the deposition of P-SYN in a blinded, prospective longitudinal study of clinically defined PD patients across a range of severity and measure the change in cutaneous P-SYN at 6-month intervals over an 18-month period.

#### Statistical considerations

Descriptive statistics will be used to report all demographic information and clinical examination findings for all groups (PD stages I-III and iRBD). For continuous variables data will be reported as mean, standard deviation, median, minimum, maximum and inter-quartile range.

For the primary objectives, the presence or absence of P-SYN will be reported as a binary outcome for the first objective (detection of P-SYN). Quantitation of P-SYN will be measured as a continuous variable for the second objective (quantitation of P-SYN). We have established that a P-SYN > 0 is defined as a ‘P-SYN positive’ result and will separate groups using dichotomous measures. We will also investigate ROC curve analysis to determine if an alternative value would maximize sensitivity and specificity using continuous quantitative measurement of P-SYN. For comparisons over time, the initial biopsy results will be compared against future biopsy results using Krippendorff’s alpha reliability estimate. Reliability will be measured by quantified continuous P-SYN values between visits with the change in continuous values used to determine mean change over time. Additional endpoints will include change in disease severity over time, or phenoconversion to clinically apparent synucleinopathy (in patients with iRBD).

Data will be compared first using repeated measures Analysis of Covariance (ANCOVA), with phenoconversion/disease progression as the predictor variable. The primary dependent variable is the quantitative measure of P-SYN deposition. Other key covariates will include sex, cognitive examination scores, disease duration, UPDRS Part 2 and Part 3 total scores, and autonomic nerve fiber density (pilomotor nerve fiber density and sudomotor nerve fiber density) (32). These will be evaluated individually and together with descriptive and multivariate statistics to identify potential violations of statistical assumptions. If normality is violated, then transformations or bootstrap methods will be used. For comparisons of demographic and laboratory data within- and between groups, nonparametric tests (Kruskal–Wallis) will be used. The GEE (general estimating equation) method will be applied if the data cannot be transformed. All data will be presented as mean ± standard deviation and interquartile range using absolute values and change from baseline data for all tests of structure and function unless otherwise specified.

#### Sample size justification

Power calculations: Power analysis is based on the minimum number of subjects across the study to reach completion required for endpoint analysis of Aims 1–2. The expected dropout rate for subjects in our 2-year study is ~ 5% per 6-month period of time based on our prior natural history data in our prior studies with DLB, multiple system atrophy and Parkinson’s disease. For Aim 1, the required number needed to obtain sufficient objective pathological data (based on prior work in patients with PD and DLB) is 15 from each group (prodromal, Hoehn and Yahr stages 1, 2 and 3). Given the data in Table 1 on P-SYN values by Hoehn and Yahr stage, we estimate 5 years to move from stage 1 to 2 and 2 to 3, suggesting a ~ 16% increase in P-SYN per year. In contrast, we noted ~37% increase in P-SYN over 6 months in a prior study of dementia with Lewy bodies, consistent with an expected accelerated disease progression compared to PD. Using the data in Tables 1, 2 we expect that over 12 months we will detect a 16% increase in P-SYN across all 4 disease stages of enrollment, but with smaller absolute changes at earlier disease stages. Given the expected 10%/year dropout and 5–10% cases that are biopsy negative we require a total of 100 subjects to complete the study with > 90% power, with a 2-sided alpha of 0.05 to detect changes in P-SYN over 12 months. By increasing study duration to 18 months, we anticipate a > 90% power, with a 2-sided alpha of 0.01 to detect changes in P-SYN over 18 months in each of the 4 study subgroups (iRBD and Hoehn and Yahr stages 1–3).

**Table 1:** P-SYN quantities by H&Y staging.

Variable	H&Y Stage 1	H&Y Stage 2	H&Y Stage 3
Number	7	70	12
P-SYN (SU’s)	1.9 ± 1.3	4.6 ± 3.8	7.9 ± 5.8

**Table 2:** High sensitivity and specificity of cutaneous P-SYN detection.

Disease	Sex (M/F)	Age (± SD)	Hoehn & Yahr stage	MOCA*	P-SYN+ (#)	P-SYN+ (%)
PD	56/40	70 ± 8	2.1 ± 0.6	25.2 ± 3.7	89	92.3%
Healthy control	7/7	59.4 ± 11.1	0 ± 0	28.1 ± 1.6	4	3.3%

*MOCA, Montreal Cognitive Assessment.

## Discussion

Over the last decade, the incidence of Parkinson’s disease has increased to 90,000 new cases per year (33). With no known preventative treatment, the increasing number of individuals with neurodegenerative disease brings increased pressure on the need for therapeutic discovery. Novel diagnostic tests can support the development of innovative therapeutics by providing an early and accurate pathological diagnosis. In addition, quantifiable measures of phosphorylated alpha-synuclein detection allow for monitoring of disease over time. This study will establish skin biopsy detection of P-SYN as an objective pathological test for synucleinopathies by quantifying phosphorylated *α*-synuclein in skin punch biopsies. The study will contribute to the development of accurate and minimally invasive diagnostic tools for early detection of PD, potentially improving patient outcomes and treatment interventions.

At present, physicians, patients, and researchers now have several tools for the detection of phosphorylated alpha synuclein. The most established methods include skin biopsy detection of P-SYN and seed amplification assay (SAA) from spinal fluid (9, 34). Both approaches offer excellent sensitivity and specificity, although skin biopsies are typically considered a less invasive procedure. Exploratory data in synuclein quantitation has been reported with skin biopsies (25, 35), but not in a longitudinal fashion. Although efforts to quantify SAA using endpoint dilution are ongoing, they have only offered limited semi-quantitative data to date (36).

The use of AI-augmented pathologic protein detection as a continuous quantitative measure of P-SYN may provide additional advantages for diagnostic evaluation. Prior studies have shown that various subtypes of synucleinopathy have different amounts of cutaneous P-SYN (25). While ordinal P-SYN quantitation measures show promise for confirming a diagnosis of a synucleinopathy, continuous quantitation measures will support efforts to establish trajectories for phenoconversion in prodromal conditions like iRBD. In addition, the natural history of P-SYN accumulation is a necessary step to define rates of change across stages of disease. This information will ultimately support studies of disease modifying therapies that target phosphorylated alpha-synuclein.

A recent report of the c-ABL kinase inhibitor, risvodetinib, in 137 patients with untreated PD has shown the potential utility of skin biopsy to quantify P-SYN over time (30). Patients were treated with 50 mg, 100 mg, or 200 mg daily doses of risvodetinib and cutaneous P-SYN was measured at baseline and 12 weeks after treatment. There were dose-dependent reductions in quantitative measures of P-SYN where reduced P-SYN was detected in 30%, 60%, and 64% of patients treated with the 50 mg, 100 mg or 200 mg doses, respectively. Although preliminary, the data strongly support the need for additional longitudinal studies to understand the natural history of peripheral P-SYN accumulation. The study also demonstrates that skin biopsy quantitation of P-SYN may be capable of detecting therapeutic changes with pharmacologic interventions. Although limited data on the use of this AI-supported P-SYN quantitation system exists, the existing study will provide direct comparison of the digital results with the direct pathologist review of the glass slides, offering an important step in the validation of this software. There are limitations to the current study. Healthy and disease control subjects are not included in this study. Numerous other studies have included both healthy and disease controls, and were considered outside of the scope of this project, given the very low rates of P-SYN detection in those populations. The duration of follow-up is limited to 18 months. If study funding can be extended, further follow-up will be considered. Finally, other synuclein biomarkers are not being tested in parallel.

## Conclusion

Despite the limitations, this will be the first prospective, blinded, natural history study of quantitative P-SYN measurement in patients with established and prodromal PD. The findings from this study could support real-time monitoring tools for Parkinson’s progression and offer significant advantages as ongoing trials with anti-synuclein therapies which currently lack a method to measure P-SYN in humans. In addition, the use of AI-enhanced pathological quantitation of P-SYN offers a scalable tool appropriate for larger volumes of testing.
